# Preoperative anemia in colorectal cancer: relationships with tumor characteristics, systemic inflammation, and survival

**DOI:** 10.1038/s41598-018-19572-y

**Published:** 2018-01-18

**Authors:** Juha P. Väyrynen, Anne Tuomisto, Sara A. Väyrynen, Kai Klintrup, Toni Karhu, Jyrki Mäkelä, Karl-Heinz Herzig, Tuomo J. Karttunen, Markus J. Mäkinen

**Affiliations:** 1 Cancer and Translational Medicine Research Unit, University of Oulu, POB 5000, Oulu, 90014 Finland; 2 Department of Pathology, Oulu University Hospital and Medical Research Center Oulu, Oulu, POB 21, Oulu, 90029 Finland; 3 Research Unit of Surgery, Anesthesia and Intensive Care, University of Oulu, POB 5000, Oulu, 90014 Finland; 4 Department of Surgery, Oulu University Hospital and Medical Research Center Oulu, POB 21, Oulu, 90029 Finland; 5 Research Unit of Biomedicine and Biocenter of Oulu, University of Oulu, POB 5000, Oulu, 90014 Finland; 6 Oulu University Hospital and Medical Research Center Oulu, POB 21, Oulu, 90029 Finland; 7 Department of Gastroenterology and Metabolism, Poznan University of Medical Sciences, ul. Szpitalna 27/33, 60-572 Poznan, Poland

## Abstract

Anemia is common in colorectal cancer (CRC) but its relationships with tumor characteristics, systemic inflammation, and survival have not been well characterized. In this study, blood hemoglobin levels and erythrocyte mean corpuscular volume (MCV) levels were measured in two independent cohorts of 148 CRC patients and 208 CRC patients, and their correlation with patient and tumor characteristics, systemic inflammatory markers (modified Glasgow Prognostic Score: mGPS; serum levels of thirteen cytokines, C-reactive protein, albumin), and survival were analyzed. We found that anemia, most frequently normocytic, followed by microcytic, was present in 43% of the patients. Microcytic anemia was most commonly associated with proximal colon tumor location. Average MCV and blood hemoglobin levels were lower in tumors with high T-class. Low blood hemoglobin associated with systemic inflammation, including high mGPS and high serum levels of C-reactive protein and IL-8. Particularly, normocytic anemia associated with higher mGPS. Normocytic anemia associated with a tendency towards worse overall survival (multivariate hazard ratio 1.61, 95% confidence interval 1.07–2.42, p = 0.023; borderline statistical significance considering multiple hypothesis testing). In conclusion, anemia in CRC patients is most frequently normocytic. Proximal tumor location is associated with predominantly microcytic anemia and systemic inflammation is associated with normocytic anemia.

## Introduction

Colorectal cancer (CRC) is one of the most common malignancies and causes of cancer deaths in the Western world^1^. CRC patients frequently have anemia at the time of the diagnosis, and anemia is one of the reasons why CRC patients enter the primary care^2^. Previously, anemia has been reported to be more common in CRC patients with tumors in proximal colon and of advanced stage^3–6^. Several studies have assessed the prognostic or predictive value of anemia in various CRC subgroups^7–20^, and reported an association between anemia and adverse outcome^8–11,13,16,17,19^. However, more data is needed, especially, of the prognostic significance of anemia relative to several important potentially confounding prognostic parameters, including tumor, node, metastasis (TNM) classification, lymphatic and venous invasion, and systemic inflammation, as well as of the prognostic value of different anemia subgroups.

Based on the erythrocyte mean corpuscular volume (MCV), anemia can be categorized as microcytic (MCV < 80 fL), normocytic (MCV 80–100 fL) or macrocytic (MCV > 100 fL)^21^. Microcytic anemia is most commonly due to iron deficiency (other, less common causes include thalassemia and anemia of chronic diseases), while the differential diagnosis of normocytic and macrocytic anemia is more diverse^21^. Of the factors associated with low MCV, thalassemias are rare in the Finnish population^22^. One of the main causes of anemia in CRC patients is blood loss to the bowel leading to iron deficiency^23^. Indeed, anemia in CRC has been reported to frequently show microcytic phenotype, especially in higher stages^3^. However, there are few studies that have assessed the relationships between clinical and histological findings of CRC and different anemia subgroups^3^.

In addition to CRC, anemia is also prevalent in other malignancies, in which no iron is lost into feces^5,7^. Anemia of inflammation, also known as anemia of chronic disease, is associated with increased circulating cytokine levels, commonly observed in infections, rheumatic and other inflammatory diseases, and cancer^24^. A proportion of CRC patients present with a systemic inflammatory response, as evidenced by increased serum levels of C-reactive protein (CRP) and decreased serum levels of albumin^25,26^. The combination of these measurements (modified Glasgow Prognostic Score, mGPS), is associated with adverse prognosis in CRC^25^. Cytokines, especially IL-6, lead to increased synthesis of CRP and decreased synthesis of albumin in the liver^27,28^. We have previously shown that CRC patients have increased serum levels of IL-6, IL-7, and IL-8 and decreased serum CCL2 levels^26,29^. Patients with advanced disease have increased serum levels of IL-1RA, IL-4, IL-6, IL-7, IL-8, CCL2, and PDGF-BB^29^. A few studies have reported that anemia is more common in CRC patients with increased mGPS^14,30^. However, to our knowledge, more detailed analyses of the relationships between anemia subgroups, serum cytokines, and other markers of systemic inflammation in CRC have not been conducted.

The objective of this study was to evaluate the determinants and clinical significance of blood hemoglobin (Hb) levels, erythrocyte MCV levels, and different anemia subgroups in two independent prospectively recruited successive cohorts of 148 CRC patients (Cohort 1) and 208 CRC patients (Cohort 2), with special emphasis on the relationships between blood Hb and the systemic inflammatory response.

## Results

### General characteristics

The average Hb level was 126.7 g/L (SD 17.3 g/L) in Cohort 1 and 126.5 g/L (SD 17.4 g/L) in Cohort 2 (Table 1), and average MCV was 88.6 fL (SD 7.0 fL) in Cohort 1 and 86.6 fL (SD 6.3 fL) in Cohort 2 (Table 1). A total of 57 (38.5%) patients in Cohort 1 and 97 (46.6%) patients in Cohort 2 had anemia (Tables S1 and S2), and it was most frequently normocytic (Cohort 1: 42 patients, 28.3%; Cohort 2: 67 patients, 32.2%), followed by microcytic (Cohort 1: 14 patients, 9.5%; Cohort 2: 29 patients, 13.9%). Only one patient in both cohorts had macrocytic anemia. Since the basic characteristics, as well as the main findings of the associations between blood Hb levels and clinicopathological variables (Tables S3 and S4), were similar in both cohorts, we combined the two cohorts for subsequent analyses, to increase the statistical power of the analyses.

**Table 1:** Characteristics of the colorectal cancer patients.

	Cohort 1 (n = 148)	Cohort 2 (n = 208)
Age, mean (SD)	66.7 (11.1)	69.2 (12.2)
**Sex**		
Male	80 (54.1%)	110 (52.9%)
Female	68 (45.9%)	98 (47.1%)
**Tumor location**		
Proximal colon	48 (32.4%)	75 (36.1%)
Distal colon	28 (18.9%)	45 (21.6%)
Rectum	72 (48.6%)	88 (42.3%)
**Preoperative radiotherapy or chemoradiotherapy**		
No	116 (78.4%)	170 (81.7%)
Yes	32 (21.6%)	38 (18.3%)
**WHO grade**		
Grade 1	21 (14.3%)	58 (28.0%)
Grade 2	108 (73.5%)	121 (58.5%)
Grade 3	18 (12.2%)	28 (13.5%)
**TNM Stage**		
Stage I	27 (18.4%)	54 (26.0%)
Stage II	54 (36.7%)	59 (28.4%)
Stage III	44 (29.9%)	71 (34.1%)
Stage IV	22 (15.0%)	24 (11.5%)
Blood hemoglobin, g/L, mean (SD)	126.7 (17.3)	126.5 (17.4)
Erythrocyte mean corpuscular volume (MCV), fL, mean (SD)	88.6 (7.0)	86.6 (6.3)

### Relationships between anemia and clinicopathological features

In the combined cohort, decreased Hb levels associated with female gender (Cohort 1: p < 0.001; Table 2). However, anemia was approximately as prevalent in male patients as in female patients (Table 3). Older patients had a tendency towards lower blood Hb levels (p = 0.0017; borderline statistical significance considering multiple hypothesis testing). Lower blood Hb associated with proximal tumor location (p < 0.001; Table 2). Particularly, microcytic anemia was common in patients with proximal colon tumors (Table 3) and average MCV was lower in subjects with proximal colon carcinomas (p < 0.001; Table 2). Preoperative RT/CRT was considered potential confounding factor. However, blood Hb levels (p = 0.986) or erythrocyte MCV levels (p = 0.636) of the rectal cancer patients who received preoperative RT/CRT did not differ from other rectal cancer patients (Table 2).

**Table 2:** Relationships between blood hemoglobin (Hb) levels (g/L), erythrocyte mean corpuscular volume (MCV) levels (fL) and clinicopathological characteristics in the combined cohort.

Variable (n)	Blood Hb, Mean (SD)	P value	Erythrocyte MCV, Mean (SD)	P value
All Patients (n = 356)	126.6 (17.3)		87.5 (6.7)	
**Age**				
<65 (n = 130)	129.5 (17.4)	0.017	87.4 (7.1)	0.908
≥65 (n = 226)	124.9 (17.1)		87.5 (6.4)	
**Sex**				
Male (n = 190)	130.8 (18.4)	<0.001	88.1 (7.0)	0.063
Female (n = 166)	121.8 (14.7)		86.8 (6.2)	
**Location of tumor**				
Proximal colon (n = 123)	116.4 (15.5)	<0.001	84.5 (7.0)	<0.001
Distal colon (n = 73)	125.1 (15.9)		86.8 (6.4)	
Rectum (n = 160)	135.1 (14.8)		90.1 (5.5)	
**Preoperative radiotherapy or chemoradiotherapy in rectal cancer patients**				
No (n = 91)	135.0 (16.0)	0.986	89.9 (5.1)	0.636
Yes (n = 69)	135.1 (13.3)		90.3 (5.9)	
**WHO grade**				
Grade 1 (n = 79)	126.3 (17.6)	0.023	87.4 (7.5)	0.582
Grade 2 (n = 229)	128.0 (17.4)		87.7 (6.3)	
Grade 3 (n = 46)	120.4 (15.7)		85.6 (6.9)	
**TNM Stage**				
Stage I (n = 81)	132.4 (17.4)	<0.001	89.7 (6.2)	<0.001
Stage II (n = 113)	122.9 (17.1)		85.9 (6.9)	
Stage III (n = 115)	128.0 (17.3)		87.8 (6.6)	
Stage IV (n = 46)	122.3 (14.8)		86.4 (6.2)	
**Primary tumor**				
T1 (n = 16)	136.4 (15.8)	<0.001	91.7 (5.7)	<0.001
T2 (n = 89)	131.5 (16.6)		89.6 (6.1)	
T3 (n = 218)	125.1 (16.9)		86.6 (6.8)	
T4 (n = 32)	119.0 (18.3)		85.8 (5.8)	
**Lymph node metastasis**				
N0 (n = 200)	126.6 (17.7)	0.135	87.5 (6.9)	0.588
N1 (n = 97)	129.0 (16.8)		87.9 (6.1)	
N2 (n = 57)	123.2 (16.7)		86.7 (6.9)	
**Distant Metastasis**				
M0 (n = 310)	127.2 (17.6)	0.072	87.6 (6.7)	0.271
M1 (n = 46)	122.3 (14.8)		86.4 (6.2)	
**Infiltrative growth pattern**				
No (n = 278)	127.1 (17.3)	0.382	87.6 (6.5)	0.475
Yes (n = 77)	125.1 (17.6)		87.0 (7.4)	
**Lymphatic invasion**				
No (n = 192)	127.0 (17.4)	0.698	87.8 (6.7)	0.303
Yes (n = 160)	126.3 (17.4)		87.1 (6.6)	
**Blood vessel invasion**				
No (n = 293)	126.9 (17.4)	0.567	87.5 (6.8)	0.867
Yes (n = 59)	125.5 (17.5)		87.4 (6.0)	
**Mismatch repair (MMR) enzyme status**				
MMR Proficient (n = 315)	127.8 (17.0)	<0.001	85.1 (6.2)	0.016
MMR Deficient (n = 40)	117.2 (17.2)		87.8 (6.7)	
**BRAF VE1 immunohistochemistry**				
Negative (n = 322)	127.3 (17.5)	0.010	87.5 (6.7)	0.234
Positive (n = 33)	119.2 (14.8)		86.1 (6.4)	
**Modified Glasgow Prognostic Score (mGPS)**				
0 (n = 269)	128.8 (17.2)	<0.001	88.1 (6.8)	0.018
1 (n = 63)	120.0 (15.8)		85.5 (6.2)	
2 (n = 8)	105.5 (5.8)		86.2 (2.7)	
**Mean corpuscular volume (MCV)**				
<80 (n = 45)	107.3 (11.0)	<0.001		
80–100 (n = 306)	129.4 (16.4)			
>100 (n = 5)	130.4 (14.0)

**Table 3:** Relationships between different categories of anemia and clinicopathological characteristics in the combined cohort.

**Variable**	No anemia (n = 202)	Microcytic anemia (n = 43)	Normocytic anemia (n = 109)	P value
**Age**				
<65	82 (63.1%)	14 (10.8%)	34 (26.2%)	0.223
≥65	120 (53.6%)	29 (12.9%)	75 (33.5%)	
**Sex**				
Male	107 (56.9%)	27 (14.4%)	54 (28.7%)	0.335
Female	95 (57.2%)	16 (9.6%)	55 (33.1%)	
**Location of tumor**				
Proximal colon	39 (31.7%)	28 (22.8%)	56 (45.5%)	<0.001
Distal colon	40 (54.8%)	10 (13.7%)	23 (31.5%)	
Rectum	123 (77.8%)	5 (3.2%)	30 (19.0%)	
**Preoperative radiotherapy or chemoradiotherapy in rectal cancer patients**				
No	69 (76.7%)	3 (3.3%)	18 (20.0%)	0.944
Yes	54 (79.4%)	2 (2.9%)	12 (17.6%)	
**WHO grade**				
Grade 1	42 (54.5%)	14 (18.2%)	21 (27.3%)	0.139
Grade 2	139 (60.7%)	23 (10.0%)	67 (29.3%)	
Grade 3	21 (45.7%)	6 (13.0%)	19 (41.3%)	
**TNM Stage**				
Stage I	53 (65.4%)	5 (6.2%)	23 (28.4%)	0.004
Stage II	50 (44.2%)	22 (19.5%)	41 (36.3%)	
Stage III	76 (66.7%)	12 (10.5%)	26 (22.8%)	
Stage IV	23 (51.1%)	4 (8.9%)	18 (40.0%)	
**Primary tumor**				
T1	12 (80.0%)	0 (0%)	3 (20.0%)	0.007
T2	59 (66.3%)	5 (5.6%)	25 (28.1%)	
T3	120 (55.3%)	33 (15.2%)	64 (29.5%)	
T4	11 (34.4%)	5 (15.6%)	16 (50.0%)	
**Lymph node metastasis**				
N0	105 (52.8%)	28 (14.1%)	66 (33.2%)	0.159
N1	65 (67.7%)	7 (7.3%)	24 (25.0%)	
N2	32 (56.1%)	8 (14.0%)	17 (29.8%)	
**Distant Metastasis**				
M0	179 (57.9%)	39 (12.6%)	91 (29.4%)	0.322
M1	23 (51.1%)	4 (8.9%)	18 (40.0%)	
**Infiltrative growth pattern**				
No	158 (57.2%)	32 (11.6%)	86 (31.2%)	0.792
Yes	44 (57.1%)	11 (14.3%)	22 (28.6%)	
**Lymphatic invasion**				
No	107 (56.0%)	24 (12.6%)	60 (31.4%)	0.894
Yes	93 (58.5%)	18 (11.3%)	48 (30.23%)	
**Blood vessel invasion**				
No	167 (57.4%)	36 (12.4%)	88 (30.2%)	0.821
Yes	33 (55.9%)	6 (10.2%)	20 (33.9%)	
**Mismatch repair (MMR) enzyme status**				
MMR Proficient	191 (61.0%)	34 (10.9%)	88 (28.1%)	<0.001
MMR Deficient	11 (27.5%)	9 (22.5%)	20 (50.0%)	
**BRAF VE1 immunohistochemistry**				
Negative	188 (58.8%)	38 (11.9%)	94 (29.4%)	0.095
Positive	13 (39.4%)	5 (15.2%)	15 (45.5%)	
**Modified Glasgow Prognostic Score (mGPS)**				
0	168 (62.9%)	30 (11.2%)	69 (25.8%)	<0.001
1	25 (39.7%)	9 (14.3%)	29 (46.0%)	
2	0 (0%)	0 (0%)	8 (100%)	

Due to the small number of macrocytic anemia cases (n = 2), macrocytic anemia category was not included in the analysis.

**Figure 1:**
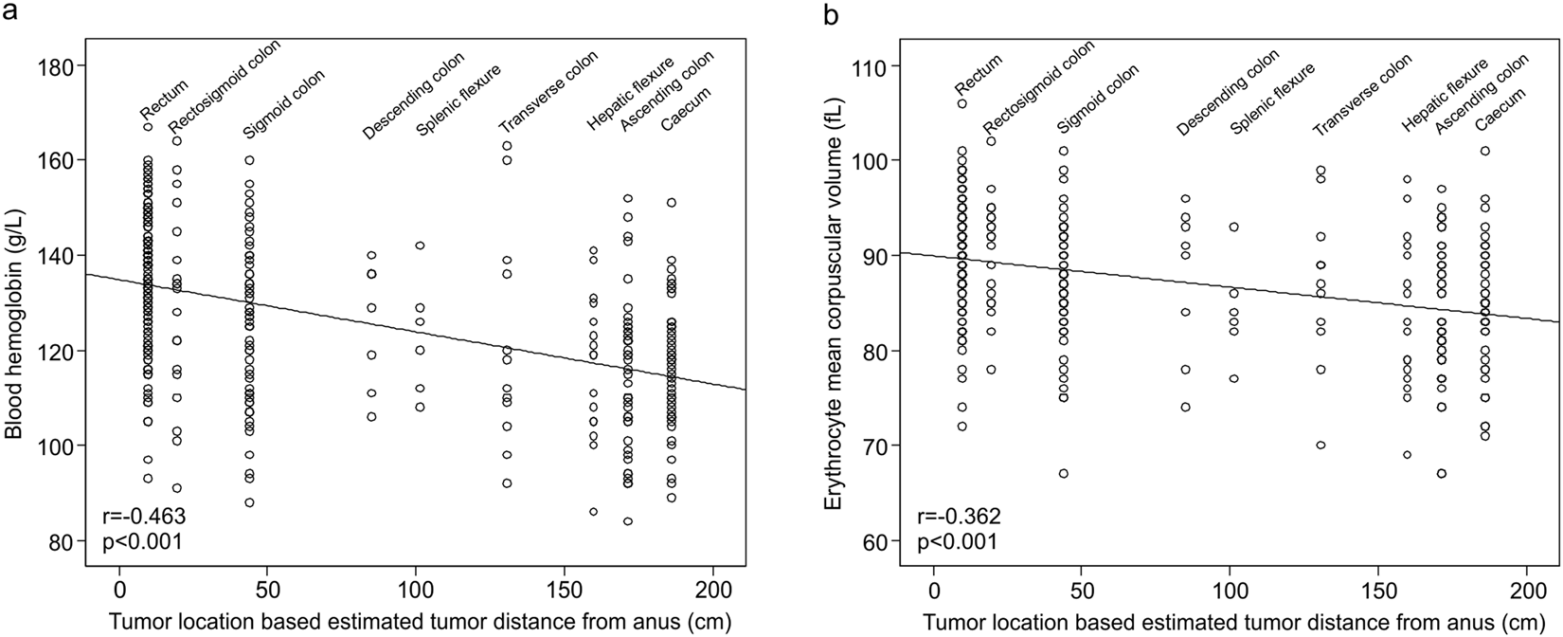
Correlation between tumor location and blood hemoglobin levels (**a**) and erythrocyte mean corpuscular volume (MCV) levels (**b**) in the combined colorectal cancer cohort (n = 356). Utilizing CT colonography data^31^, tumor location was coded into an estimation of tumor distance from anus. This estimation had linear negative correlation with blood hemoglobin levels and erythrocyte MCV levels.

To study the association between tumor location and blood Hb levels in more detail, we recoded the tumor location into a continuous variable, based on average distance of each subsite to anus, utilizing recent computed tomography colonography data^31^. This approach has been successfully applied previously^32^. This estimate (tumor location based estimated tumor distance from anus) had moderate negative correlation with blood hemoglobin levels (p < 0.001) and erythrocyte MCV levels (p < 0.001; Fig. 1).

Of the other clinicopathological variables, decreased Hb levels associated with advanced TNM stage (p < 0.001), especially higher T-class (p < 0.001; Table 2). Average MCV was lower in tumors with higher T-class (p < 0.001; Table 2), suggesting higher prevalence of iron deficiency in these patients.

Both BRAF mutation and MMR deficiency are molecular features commonly associated with serrated pathway of CRC and have been reported to be common in tumors in proximal colon^33^. We therefore hypothesized that these molecular features could associate with anemia in CRC. Indeed both microcytic and normocytic anemia were more common in the patients with MMR deficient tumors relative to the patients with MMR proficient tumors (p < 0.001; Table 3). BRAF mutation (p = 0.010; borderline statistical significance considering multiple hypothesis testing) associated with a tendency towards lower Hb levels, while MMR deficiency associated with significantly lower blood Hb (p < 0.001; Table 2). However, when the analysis was restricted to the tumors in proximal colon (Table S5), there were no significant associations between anemia and MMR enzyme status (p = 0.577) or BRAF mutation (p = 0.885), suggesting that the association between proximal tumor location and MMR deficiency (and BRAF mutation) could mainly account for the observed association between MMR deficiency and anemia.

There were no significant associations between blood Hb levels and infiltrative tumor growth, lymphatic invasion or blood vessel invasion.

### Relationships between anemia, serum cytokine levels, and systemic inflammation

A major hypothesis of the study was that anemia would be associated with systemic inflammation in CRC. Supporting the hypothesis, blood Hb negatively correlated with mGPS (p < 0.001; Table 2) and serum C-reactive protein (univariate p < 0.001; tumor stage and location and patient gender adjusted p = 0.012; borderline statistical significance considering multiple hypothesis testing; Table S6) and positively correlated with serum albumin (univariate p < 0.001; tumor stage and location and patient gender adjusted p < 0.001). Higher mGPS associated with predominantly normocytic anemia (p < 0.001; Table 3).

**Figure 2:**
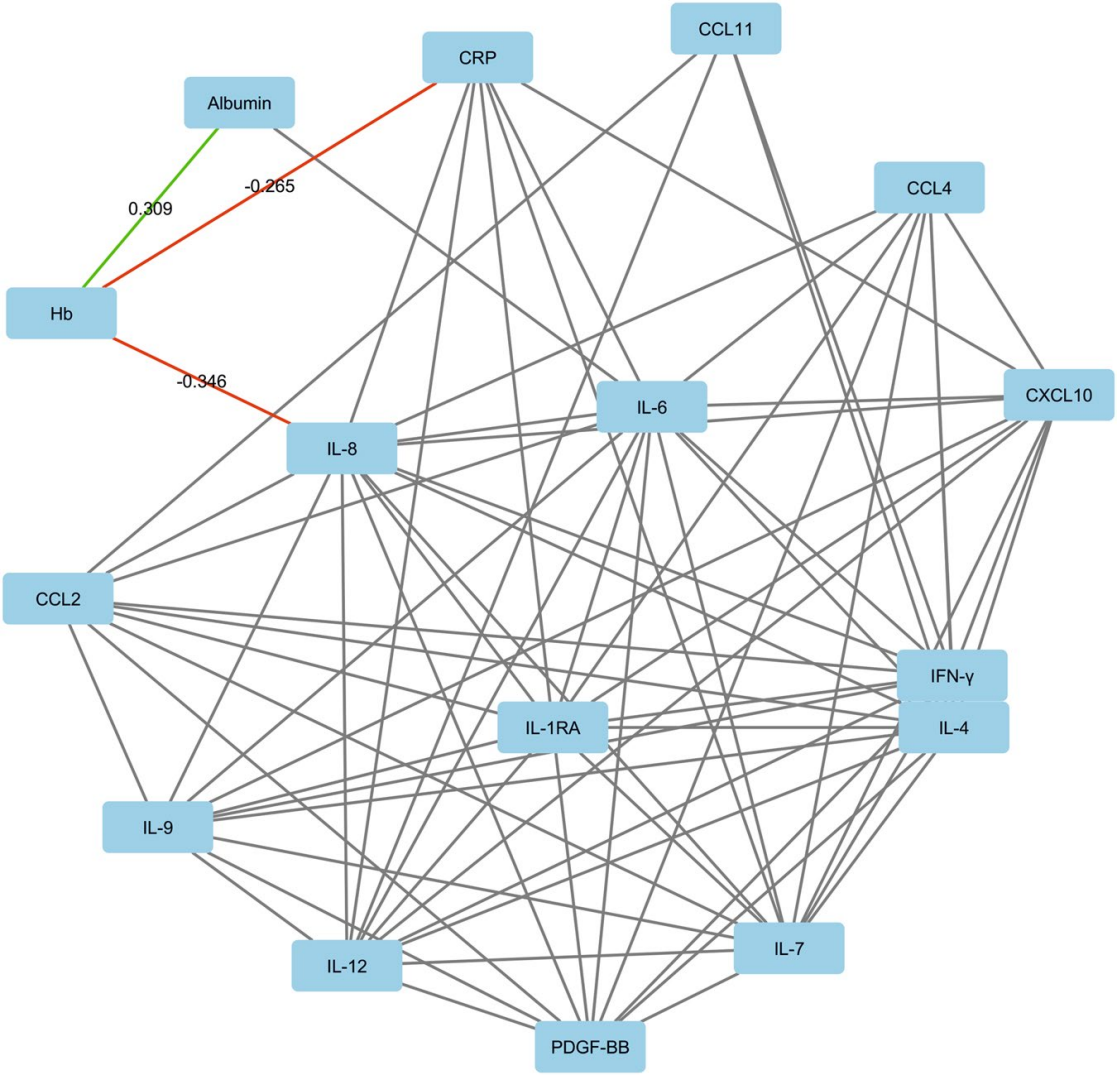
2D visualization of the relationships between blood hemoglobin (Hb), serum C-reactive protein (CRP) levels, serum albumin levels, and serum cytokine levels in Cohort 1. The edges (connecting lines) depict the associations between the variables (only those with p < 0.0015 shown). The edge length illustrates the significance of the association. The correlations between Hb and other variables are represented by green (positive correlation) and red (negative correlation) edges, with the label indicating corresponding Pearson r for the correlation. The other associations are indicated by the grey edges. The 2D visualization was created with Cytoscape software platform^55^, utilizing the Prefuse force directed algorithm weighted by the statistical significances of the correlations between individual variables. Abbreviations: CCL: Chemokine (C-C motif) ligand; CRP: C-reactive protein; CXCL: Chemokine (C-X-C motif) ligand; Hb: Hemoglobin; IFN: interferon IL: interleukin; PDGF: Platelet-derived growth factor.

Serum analysis of thirteen cytokines was conducted in Cohort 1, and blood Hb negatively correlated with serum IL-8 (univariate p < 0.001; tumor stage and location and patient gender adjusted p = 0.009; borderline statistical significance considering multiple hypothesis testing; Table S7; Fig. 2). Normocytic anemia was associated with increased serum levels of CRP (p = 0.003; borderline statistical significance considering multiple hypothesis testing; Table S8), and IL-8 (p = 0.001), and decreased serum levels of albumin (p < 0.001), while microcytic anemia did not show significant associations with serum CRP, albumin, or cytokines (Table S8).

### Multiple linear regression model for blood hemoglobin levels

A multiple linear regression model was constructed to evaluate the individual contribution of different explanatory variables, assessed in univariate analyses in Table 2 and S6, to blood Hb levels (Table 4). The model indicated that female gender, distal tumor location, higher T-class, and lower serum albumin levels independently associated with decreased blood Hb levels in CRC.

**Table 4:** Multiple linear regression model for blood hemoglobin levels in the combined cohort.

Variable	Beta	p value
Patient age	−0.107	0.023
Patient gender (male vs. female)	−0.163	<0.001
Tumor location based estimated tumor distance from anus	−0.384	<0.001
T classification (ordinal categorical; T1, T2, T3, T4)	−0.165	<0.001
N classification (N0 vs. N1–2)	0.073	0.142
M classification (M0 vs. M1)	−0.033	0.507
Mismatch repair (MMR) enzyme status (deficient vs. proficient)	−0.021	0.688
Serum C-reactive protein	−0.083	0.080
Serum albumin	0.229	<0.001

Serum C-reactive protein was logarithmically transformed because of positive skewness. R^2^ = 0.359.

### Survival analyses

Finally, survival analyses were carried out to evaluate the prognostic value of blood Hb levels in CRC. In the univariate analyses (Table 5), high blood Hb (≥140 g/L) associated with a tendency towards improved CSS (HR = 0.37, 95% CI = 0.19–0.75; p = 0.005; borderline statistical significance considering multiple hypothesis testing) and OS (HR = 0.46, 95% CI = 0.27–0.78; p = 0.004; borderline statistical significance considering multiple hypothesis testing) and normocytic anemia associated with a tendency towards poor OS (HR = 1.69, 95% CI = 1.15–2.48; p = 0.007; borderline statistical significance considering multiple hypothesis testing). Of these, the association between normocytic anemia and poor OS was highest in multivariate Cox regression models, but it was borderline statistical significance due to multiple hypothesis testing (HR = 1.61, 95% CI = 1.07–2.42, p = 0.023; Table 6).

**Table 5:** Univariate analysis of time to recurrence (TTR), cancer-specific survival (CSS), and overall survival (OS) according to blood hemoglobin levels with different cut-off points, erythrocyte mean corpuscular volume (MCV), and different anemia categories.

Variable	TTR^A^			CSS^B^			OS^C^		
	HR	95% CI	p value	HR	95% CI	p value	HR	95% CI	p value
**Cohort 1**									
**Blood hemoglobin**									
<110 g/L vs. ≥ 110 g/L	0.79	0.43–1.45	0.444	0.66	0.39–1.12	0.119	0.64	0.42–0.99	0.045
<120 g/L vs. ≥ 120 g/L	1.39	0.79–2.45	0.257	0.70	0.44–1.11	0.132	0.65	0.44–0.95	0.026
<130 g/L vs. ≥ 130 g/L	1.30	0.79–2.13	0.301	0.61	0.38–0.97	0.037	0.59	0.40–0.87	0.008
<140 g/L vs. ≥ 140 g/L	1.06	0.62–1.84	0.829	0.37	0.19–0.75	0.005	0.46	0.27–0.78	0.004
**Erythrocyte mean corpuscular volume (MCV)**									
<80 fL vs. ≥ 80 fL	1.14	0.54–2.40	0.726	1.16	0.58–2.32	0.682	1.28	0.70–2.33	0.422
<90 fL vs. ≥ 90 fL	1.09	0.67–1.80	0.722	0.91	0.57–1.44	0.683	1.12	0.77–1.64	0.542
**Anemia**									
No vs. Yes	0.74	0.44–1.24	0.252	1.26	0.80–1.98	0.320	1.44	0.99–2.10	0.055
**Microcytic anemia**									
No vs. Yes	0.91	0.43–1.91	0.798	0.80	0.38–1.66	0.546	0.75	0.40–1.40	0.363
**Normocytic anemia**									
No vs. Yes	0.67	0.36–1.23	0.194	1.39	0.87–2.24	0.171	1.69	1.15–2.48	0.007

^A^n = 299; median follow-up time 51.2 months (IQR 24.7–71.4); 63 (21.0%) events; 57 (16.0%) cases excluded from the analysis because the operation was not radical or no follow-up data available.

^B^n = 354; median follow-up time 56.0 months (IQR 32.9–78.6); 75 (21.2%) events; 2 (0.6%) cases excluded from the analysis because no follow-up data available.

^C^n = 356; median follow-up time 56.0 months (IQR 32.9–78.6); 110 (30.9%) events.

Abbreviations: CI: confidence interval; CSS: cancer specific survival; HR: hazard ratio; OS: overall survival; TTR: time to recurrence.

**Table 6:** Cox proportional hazard regression models for time to recurrence (TTR), cancer-specific survival (CSS), and overall survival (OS) according to normocytic anemia and clinicopathological characteristics.

	TTR			CSS			OS		
	HR	95% CI	p value	HR	95% CI	p value	HR	95% CI	p value
Tumor invasion (T1-T2 vs. T3-T4)	1.92	0.94–3.91	0.073	1.26	0.65–2.44	0.495	1.10	0.67–1.80	0.720
Nodal metastases (N0 vs. N1-N2)	5.25	2.89–9.56	<0.001	4.08	2.15–7.75	<0.001	2.34	1.49–3.68	<0.001
Distant metastases (M0 vs. M1)	—	—	—	6.77	3.94–11.6	<0.001	4.30	2.68–6.89	<0.001
Tumor location (Colon vs. Rectum)	1.46	0.78–2.74	0.236	0.93	0.54–1.60	0.791	0.91	0.57–1.44	0.679
Preoperative radiotherapy or chemoradiotherapy (No vs. Yes)	0.96	0.48–1.89	0.896	1.03	0.51–2.09	0.930	0.97	0.54–1.76	0.928
Normocytic anemia (No vs. Yes)	0.89	0.47–1.72	0.733	1.36	0.82–2.27	0.239	1.61	1.07–2.42	0.023
mGPS (0 vs. 1–2)	0.63	0.27–1.49	0.292	1.31	0.76–2.26	0.331	1.40	0.89–2.19	0.145

The models aimed to enlighten the prognostic value of normocytic anemia in CRC, relative to TNM variables and systemic inflammation (mGPS). Abbreviations: CI: confidence interval; CSS: cancer specific survival; HR: hazard ratio; mGPS: modified Glasgow Prognostic Score; OS: overall survival; TTR: time to recurrence.

## Discussion

To our knowledge, this is so far the most extensive study on the relationships between blood Hb and erythrocyte MCV, CRC patient characteristics, tumor histopathological and molecular features, and survival in CRC. The main findings indicate that decreased blood Hb, and especially normocytic anemia, in CRC is associated with systemic inflammation, while low MCV and microcytic anemia are associated with advanced T-class and proximal tumor location. These findings indicate that in CRC, systemic inflammatory effects are important determinants of blood Hb. However, also the tumor burden itself manifests in anemia and in decrease of red blood cell size.

We found significantly lower blood Hb in patients with tumors in proximal colon relative to distal colon and rectum. This confirms the results of several previous studies^3–5^. Fecal occult blood screening has been reported to have high sensitivity for the detection of both colon and rectal tumors^34^, indicating that both colon and rectal cancers frequently bleed into the lumen. The differences between proximal and distal CRC can be mechanistically related to the bleeding, but also other effects – e.g. immunological mechanisms – are needed to be taken into account.

The tumors in the proximal colon often have distinct characteristic genetic properties (particularly, BRAF V600E mutation and MMR deficiency), resulting from the development of serrated precursor lesions through the serrated route of colorectal carcinogenesis^33^. Interestingly, the patients with MMR deficient tumors had a very high prevalence of anemia (72.5%), and both microcytic and normocytic anemia were overrepresented in MMR deficient subgroup. However, MMR deficient tumors are mostly located in proximal colon, and our subsequent analyses indicated that there was no significant difference in the prevalence of different anemia subtypes between patients with MMR deficient and MMR proficient tumors in proximal colon. Moreover, MMR deficiency was not a significant predictor of blood hemoglobin levels in multiple linear regression. Nevertheless, the number of MMR deficient cases in this study was rather small (n = 40), and further research is required to reliably analyze blood Hb levels in MMR deficient cases of different tumor locations.

Our results indicate that blood Hb levels in CRC inversely associate with systemic inflammation. High mGPS, high serum IL-8, and low serum albumin, particularly, associated with normocytic anemia. IL-8 is a proinflammatory chemokine associated with the promotion of neutrophil chemotaxis and degranulation^35^. Serum IL-8 levels are increased in many malignancies, including CRC^29^, and IL-8 is considered an important contributor of cancer-associated inflammation^35^. Serum albumin levels depict systemic inflammation, since the synthesis of albumin decreases as a response to IL-6^25,28^. Overall, these findings support the idea that, especially, normocytic anemia in CRC is associated with the systemic inflammation. This association might also have therapeutic significance, since the modulation of the inflammatory response has shown some promise in the treatment of the anemia of inflammation^36^.

The reported mechanisms linking inflammation and anemia are diverse^24^. First, pro-inflammatory cytokines including IL-6 stimulate the hepatic expression of hepcidin, which inhibits the absorption of iron in the duodenum^37^. Second, in inflammatory conditions, there is limited availability of iron for erythroid cells, due to the alterations in macrophage functions^24^. Cytokine stimulus leads to the activation of macrophages, which phagocytose and degrade erythrocytes. IFN-γ stimulates the uptake of iron by macrophages by increasing the expression of divalent metal transporter 1, and IL-10 stimulates the uptake of transferrin-bound iron by upregulating transferrin receptor expression^24^. Several cytokines, including IL-6 and IL-10 induce ferritin expression and stimulate the storage and retention of iron within macrophages^24^. Third, several cytokines, including TNF-α and IFNγ, inhibit the synthesis of erythropoietin in the kidney, leading to diminished erythropoiesis^38^. Fourth, pro-inflammatory cytokines, such as TNF-α and IFNγ directly inhibit the proliferation of erythroid progenitor cells^24^. Against our hypotheses, our results did not indicate significant correlations between blood Hb levels and these cytokines, including IL-6 and IFNγ.

The earlier studies on the prognostic significance of blood Hb levels in CRC have been controversial, with reports of the association of anemia with lower survival in advanced colorectal cancer^9^, lower OS in stage II-III CRC treated with FOLFOX chemotherapy^10^, lower OS in stage I-III CRC^13^, no independent prognostic value in stage I-III CRC^14^, adverse OS in metastasized CRC^15,19^, poor OS in stage I-III rectal cancer, and no prognostic value in unselected colon cancer material^20^. Moreover, iron deficiency anemia has been associated with diminished disease-free survival in T3N0M0 colon cancer^8^. In the univariate analyses of our study, anemia did not significantly associate with disease outcome. Instead, normocytic anemia associated with a trend towards adverse OS in both univariate and multivariate survival analyses (multivariate HR = 1.61, 95% CI = 1.07–2.42, p = 0.023; borderline statistical significance due to multiple hypothesis testing). In the multivariate Cox regression model, the significance of normocytic anemia was superior to mGPS. Earlier studies have established mGPS as an independent additional prognostic parameter in CRC^25^, and the relatively small number of cases in the analyses could have affected the results of this study. Relying solely on Bonferroni corrected p values could increase the risk of type 2 statistical error^39^. Therefore, this result encourages further studies to assess the prognostic significance of normocytic anemia, especially in relation to mGPS and other systemic inflammatory biomarkers, in larger cohorts. Moreover, the limited sample size in our study does not allow sensible subgroup analysis in, *e.g*., stage II patients, which would be required for firm conclusions on the prognostic value of blood Hb in these different patient subgroups.

In addition to the relatively low numbers of specific patient groups, such as MMR deficient cases, additional limitations need to be considered in the interpretation of the results. First, MCV was used in the categorization of anemia. While reduced MCV is relatively specific for iron deficiency, its sensitivity is lower than, *e.g*., that of serum transferrin receptor, especially in the presence of chronic diseases such as CRC^40^. For this study, no additional markers for iron deficiency were available, and further characterization of anemia using additional parameters would be beneficial in subsequent studies. Second, no data was available on the preoperative iron supplementation of the patients. However, current available data do not provide conclusive evidence on preoperative iron supplements significantly affecting blood Hb in patients undergoing surgery for CRC^41^. Preoperative RT/CRT was considered another potential confounding factor but the Hb levels of the patients who received preoperative RT/CRT did not significantly differ from those of the other rectal cancer patients. Multiple hypotheses were tested in this observational study. However, we adjusted the level of statistical significance to p = 0.0015 (≈0.05/34) by Bonferroni correction and interpreted the results with p = 0.05–0.0015 (considered borderline statistical significance) cautiously. This approach could result in some increase in type 2 statistical error but reduce the risk of type 1 error. The strengths in the study was the inclusion of two independent well-characterized, prospectively recruited study cohorts. The broad array of analyzed tumor characteristics and systemic inflammatory markers enabled us to investigate their relative significance for blood Hb levels. In addition to extensive characterization of the associations between blood Hb and systemic inflammation, this is, to our knowledge, the first study to analyze the correlations between different anemia subgroups in CRC and MMR enzyme status and BRAF mutation.

In conclusion, anemia is common in CRC patients and it is most frequently normocytic followed by microcytic. Proximal tumor location is preferentially associated with microcytic anemia, while systemic inflammation is associated with normocytic anemia. Further data is needed on the prognostic value of anemia in different patient subgroups.

## Methods

### Patients

This study was introduced to all newly diagnosed CRC patients operated in Oulu University Hospital in 2006–2014, of which the patients who signed an informed consent to participate were included. The patients with earlier or simultaneously diagnosed other malignant diseases were excluded. The study includes two independent, consecutive, prospectively recruited cohorts of CRC patients. Cohort 1 is an earlier described cohort of 148 CRC patients operated in Oulu University Hospital in 2006–2010 (Table 1)^29,42,43^, with up to 120-month follow-up data (Time to recurrence, TTR; cancer specific survival, CSS; overall survival, OS) collected from the clinical records and from Statistics Finland^44–46^. Cohort 2 consists of 208 CRC patients operated in Oulu University Hospital in 2010–2014, with up to 60-month follow-up data (TTR, CSS, OS) collected from the clinical records and from Statistics Finland (Table 1). TTR was defined as time from the operation to the recurrence of the same cancer, CSS was defined as time from the operation to death from the same cancer, and OS was defined as time from the operation to death, irrespective of cause. Tumor location data, acquired from the clinical records was recoded into a continuous variable, based on average distance of each subsite to anus, utilizing recent computed tomography colonography data^31^: rectum 9.75 cm, rectosigmoid colon 19.5 cm, sigmoid colon 44 cm, descending colon 85 cm, splenic flexure 101,5 cm, transverse colon 130,65 cm, hepatic flexure 159,8 cm, ascending colon 171,35 cm, and caecum 187,25 cm. This approach has been successfully applied previously^32^. In both cohorts, the preoperative staging of rectal cancer was performed with magnetic resonance imaging, and the patients with cT3 or cT4 rectal tumors (Cohort 1: n = 32, 21.6%; Cohort 2: n = 38, 18.3%) received preoperative radiotherapy or chemoradiotherapy (RT/CRT). The study was performed with the approval of the Ethics Committee of Oulu University Hospital (58/2005, 184/2009) and in accordance with the Declaration of Helsinki. All the patients and the controls had signed an informed consent to participate. The REporting recommendations for tumor MARKer prognostic studies (REMARK) were taken into account in the study design and reporting^47^.

### Blood analyses

Preoperative blood and serum samples from the patients were collected^29^. In both cohorts, blood Hb levels, erythrocyte MCV levels, serum CRP levels and serum albumin levels were measured in the laboratory of Oulu University Hospital^29,42^. Anemia was defined according to WHO criteria as blood Hb levels < 120 g/L in women or < 130 g/L in men^48^. It was classified according to erythrocyte MCV levels as microcytic (MCV < 80 fL), normocytic (MCV 80–100 fL), and macrocytic (MCV > 100 fL). mGPS was determined according to the established criteria (mGPS0: serum CRP ≤ 10 mg/L and serum albumin ≥ 35 g/L or < 35 g/L; mGPS1: serum CRP > 10 mg/L and serum albumin ≥ 35 g/L; mGPS2: serum CRP > 10 mg/L and serum albumin < 35 g/L)^25,49^. In Cohort 1, the serum analysis of 27 cytokines was performed with Bio-Plex Pro Human pre-manufactured 27-Plex Cytokine Panel (Bio-Rad, Hercules, CA, USA), as described earlier^29^. As described earlier in more detail, 14 cytokines had many values below or above the assay detection limits, and therefore, 13 cytokines (IL-1ra, IL-4, IL-6, IL-7, IL-8, IL-9, IL-12, IFN-γ, CXL10, CCL2, CCL4, CCL11, and PDGF-BB) with less than four values outside the assay working range were included in this study^29^.

### Histopathological analysis

The staging of the tumors was conducted according to TNM6 (Cohort 1) or TNM7 (Cohort 2) and the grading according to the World Health Organization (WHO) criteria (both cohorts). Lymphatic invasion was defined as tumor cells present in vessels with an endothelial lining but lacking a muscular wall, and blood vessel invasion was evaluated positive if there were tumor cells in vessels with a thick muscular wall or in vessels containing red blood cells^50^. Tumor growth pattern at the tumor border was classified using the earlier described criteria as infiltrative or expanding^50,51^. All the histological analyses were performed blinded to the clinical data.

### Immunohistochemistry

For both cohorts, tissue microarrays were utilized in immunohistochemical analyses. For both cohorts, the arrays included 1–4 cores of 3.0 mm diameter (Cohort 1, median 3; Cohort 2, median 4), depending on the size of the tumor, from the invasive margin and the tumor center^52^. Immunohistochemistry for mismatch repair (MMR) enzymes MLH1, MSH2, MSH6, and PMS2 was conducted, as described earlier, to evaluate MMR enzyme status^42,53^. BRAF V600E specific VE1 immunohistochemistry was conducted with Ventana Bench-Mark XT immunostainer (Ventana Medical Systems, Tucson, AZ)^54^, to evaluate BRAF mutation status of both cohorts. Our earlier study has indicated that the method had a sensitivity of 100% and a specificity of 99.3% in detecting BRAF V600E mutation^54^.

### Statistical analyses

The statistical analyses were conducted using IBM SPSS Statistics for Windows, Version 22.0 (IBM Corp., Armonk, NY). Pearson correlation coefficients (r) were used to determine the correlation between two continuous variables. To normalize their distribution, logarithmic transformation was applied to variables with positive skewness. The statistical significances of the associations between categorical and continuous variables were analyzed by independent samples t-test or Mann-Whitney test (comparing two classes), or one-way analysis of variances (ANOVA) or Kruskal-Wallis test (comparing three or more classes), while the statistical significances of the associations between two categorical variables were analyzed with χ^2^ test of Fisher exact test, as appropriate. A multiple linear regression analysis of the correlation of blood Hb levels with selected clinicopathological factors was conducted. Cytoscape, an open source software platform for visualizing complex networks, was used in creating a 2D visualization of the relationships between blood Hb levels and serum levels of systemic inflammatory markers with the Prefuse force directed algorithm weighted by the statistical significances of the correlations between individual variables^55^. The survival outcomes of the patient subgroups were analyzed with Kaplan-Meier method, log-rank tests, and Cox regression analysis. All p values are two-tailed. We assessed the associations between blood Hb levels and 34 parameters. Due to multiple hypothesis testing, we adjusted the level of statistical significance to p = 0.0015 (≈0.05/34) by Bonferroni correction, regarded the results with p = 0.05–0.0015 as of borderline statistical significance, and interpreted the results cautiously.

### Data availability statement

All data generated or analyzed during this study are available from the corresponding author on reasonable request.

## Electronic supplementary material

Supplementary online material
