# Supplementary material for: Preoperative anemia in colorectal cancer: relationships with tumor characteristics, systemic inflammation, and survival
Source: Sci Rep. 2018 Jan 18;8:1126. doi: 10.1038/s41598-018-19572-y (PMC5773501; doi:10.1038/s41598-018-19572-y)
Supplement: Supplementary file 1 — Supplementary online material [file 41598_2018_19572_MOESM1_ESM.pdf]

Supplementary online material

Preoperative anemia in colorectal cancer: relationships with tumor characteristics, systemic inflammation, and survival

Juha P. Väyrynen[1], Anne Tuomisto[1], Sara A. Väyrynen[1], Kai Klintrup[2], Toni Karhu[3], Jyrki Mäkelä[2], Karl-Heinz Herzig[3,4], Tuomo J. Karttunen[1], Markus J. Mäkinen[1]

[1] Cancer and Translational Medicine Research Unit, University of Oulu, POB 5000, Oulu 90014, Finland; Finland; Department of Pathology, Oulu University Hospital and Medical Research Center Oulu, Oulu, POB 21, Oulu 90029, Finland

[2] Research Unit of Surgery, Anesthesia and Intensive Care, University of Oulu, POB 5000, Oulu 90014, Finland; Department of Surgery, Oulu University Hospital and Medical Research Center Oulu, POB 21, Oulu 90029, Finland

[3] Research Unit of Biomedicine and Biocenter of Oulu, University of Oulu, POB 5000, Oulu 90014, Finland; Oulu University Hospital and Medical Research Center Oulu, POB 21, Oulu 90029, Finland

[4] Department of Gastroenterology and Metabolism, Poznan University of Medical Sciences, ul. Szpitalna 27/33, 60-572, Poznan, Poland

Supplementary online material

Table S1. Relationships between different categories of anemia and clinicopathological characteristics in Cohort 1.

**Variable**	No anemia (n=91)	Microcytic anemia (n=14)	Normocytic anemia (n=42)	P value
**Age**				
<65	38 (64.4%)	4 (6.8%)	17 (28.8%)	0.668
≥65	53 (60.2%)	10 (11.4%)	25 (28.4%)	
**Sex**				
Male	50 (63.3%)	8 (10.1%)	21 (26.6%)	0.848
Female	41 (60.3%)	6 (8.8%)	21 (30.9%)	
**Location of tumor**				
Proximal colon	18 (37.5%)	11 (22.9%)	19 (39.6%)	<0.001
Distal colon	15 (53.6%)	2 (7.1%)	11 (39.3%)	
Rectum	58 (81.7%)	1 (1.4%)	12 (16.9%)	
**Preoperative radiotherapy or chemoradiotherapy**				
No	64 (55.2%)	14 (12.1%)	38 (32.8%)	0.004
Yes	27 (87.1%)	0 (0%)	4 (12.9%)	
**WHO grade**				
Grade 1	14 (70.0%)	2 (10.0%)	4 (20.0%)	0.871
Grade 2	67 (62.0%)	10 (9.3%)	31 (28.7%)	
Grade 3	10 (55.6%)	2 (11.1%)	6 (33.3%)	
**TNM Stage**				
Stage I	22 (81.5%)	1 (3.7%)	4 (14.8%)	0.038
Stage II	26 (48.1%)	6 (11.1%)	22 (40.7%)	
Stage III	31 (72.1%)	5 (11.6%)	7 (16.3%)	
Stage IV	12 (54.5%)	2 (9.1%)	8 (36.4%)	
**Primary tumor**				
T1	5 (100.0%)	0 (0%)	0 (0%)	0.031
T2	23 (79.3%)	1 (3.4%)	5 (17.2%)	
T3	60 (59.4%)	12 (11.9%)	29 (28.7%)	
T4	3 (27.3%)	1 (9.1%)	7 (63.6%)	
**Lymph node metastasis**				
N0	49 (57.6%9	8 (9.4%)	28 (32.9%)	0.131
N1	28 (80.0%)	2 (5.7%)	5 (14.3%)	
N2	14 (56.0%)	4 (16.0%)	7 (28.0%)	
**Distant Metastasis**				
M0	79 (63.2%)	12 (9.6%)	34 (27.2%)	0.783
M1	12 (54.5%)	2 (9.1%)	8 (36.4%)	
**Infiltrative growth pattern**				
No	63 (61.2%)	9 (8.7%)	31 (30.0%)	0.645
Yes	28 (65.1%)	5 (11.6%)	10 (23.3%)	
**Lymphatic invasion**				
No	50 (60.2%)	7 (8.4%)	26 (31.3%)	0.756
Yes	39 (65.0%)	6 (10.0%)	15 (25.0%)	

**Blood vessel invasion**				
No	70 (60.3%)	12 (10.3%)	34 (29.3%)	0.490
Yes	19 (70.4%)	1 (3.7%)	7 (25.9%)	
**Mismatch repair (MMR) enzyme status**				
MMR Proficient	88 (65.2%)	12 (8.9%)	35 (25.9%)	0.034
MMR Deficient	3 (27.3%)	2 (18.2%)	6 (54.5%)	
**BRAF VE1 immunohistochemistry**				
Negative	85 (64.4%)	11 (8.3%)	36 (27.3%)	0.063
Positive	5 (35.7%)	3 (21.4%)	6 (42.9%)	
**Modified Glasgow Prognostic Score (mGPS)**				
0	79 (67.5%)	12 (10.3%)	26 (22.2%)	0.002
1	12 (48.0%)	2 (8.0%)	11 (44.0%)	
2	0 (0%)	0 (0%)	5 (100.0%)	

Due to the small number of macrocytic anemia cases (Cohort 1: n=1, Cohort 2: n=1), macrocytic anemia category was not included in the analysis.

Table S2. Relationships between different classes of anemia and clinicopathological characteristics in Cohort 2.

Variable	No anemia (n=111)	Microcytic anemia (n=29)	Normocytic anemia (n=67)	P value
**Age**				
<65	44 (62.0%)	10 (14.1%)	17 (23.9%)	0.150
≥65	67 (49.3%)	19 (14.0%)	50 (36.8%)	
**Sex**				
Male	57 (52.3%)	19 (17.4%)	33 (30.3%)	0.334
Female	54 (55.1%)	10 (10.2%)	34 (34.7%)	
**Location of tumor**				
Proximal colon	21 (28.0%)	17 (22.7%)	37 (49.3%)	<0.001
Distal colon	25 (55.6%)	8 (17.8%)	12 (26.7%)	
Rectum	65 (74.7%)	4 (4.6%)	18 (20.7%)	
**Preoperative radiotherapy or chemoradiotherapy**				
No	83 (49.1%)	27 (16.0%)	59 (34.9%)	0.017
Yes	28 (73.7%)	2 (5.3%)	8 (21.1%)	
**WHO grade**				
Grade 1	28 (49.1%)	12 (21.1%)	17 (29.8%)	0.132
Grade 2	72 (59.5%)	13 (10.7%)	36 (29.8%)	
Grade 3	11 (39.3%)	4 (14.3%)	13 (46.4%)	
**TNM Stage**				
Stage I	31 (57.4%)	4 (7.4%)	19 (35.2%)	0.017
Stage II	24 (40.7%)	16 (27.1%)	19 (32.2%)	
Stage III	45 (63.4%)	7 (9.9%)	19 (26.8%)	
Stage IV	11 (47.8%)	2 (8.7%)	10 (43.5%)	
**Primary tumor**				
T1	7 (70.0%)	0 (0%)	3 (30.0%)	0.204
T2	36 (60.0%)	4 (6.7%)	20 (33.3%)	
T3	60 (51.7%)	21 (18.1%)	35 (30.2%)	
T4	8 (38.1%)	4 (19.0%)	9 (42.9%)	
**Lymph node metastasis**				
N0	56 (49.1%)	20 (17.5%)	38 (33.3%)	0.461
N1	37 (60.7%)	5 (8.2%)	19 (31.1%)	
N2	18 (56.3%)	4 (12.5%)	10 (31.3%)	
**Distant Metastasis**				
M0	100 (54.3%)	27 (14.7%)	57 (31.0%)	0.417
M1	11 (47.8%)	2 (8.7%)	10 (43.5%)	
**Infiltrative growth pattern**				
No	95 (54.9%)	23 (13.3%)	55 (31.8%)	0.681
Yes	16 (47.1%)	6 (17.6%)	12 (35.3%)	
**Lymphatic invasion**				
No	57 (52.8%)	17 (15.7%)	34 (31.5%)	0.775
Yes	54 (54.5%)	12 (12.1%)	33 (33.3%)	
**Blood vessel invasion**				
No	97 (55.4%)	24 (13.7%)	54 (30.9%)	0.472

Yes	14 (43.8%)	5 (15.6%)	13 (40.6%)	
**Mismatch repair (MMR) enzyme status**				
MMR Proficient	103 (57.9%)	22 (12.4%)	53 (29.8%)	0.008
MMR Deficient	8 (27.6%)	7 (24.1%)	14 (48.3%)	
**BRAF VE1 immunohistochemistry**				
Negative	103 (54.8%)	27 (14.4%)	58 (30.9%)	0.368
Positive	8 (42.1%)	2 (10.5%)	9 (47.4%)	
**Modified Glasgow Prognostic Score (mGPS)**				
0	89 (59.3%)	18 (12.0%)	43 (28.7%)	0.006
1	13 (34.2%)	7 (18.4%)	18 (47.4%)	
2	0 (0%)	0 (0%)	3 (100.0%)	

Due to the small number of macrocytic anemia cases (Cohort 1: n=1, Cohort 2: n=1), macrocytic anemia category was not included in the analysis.

Table S3. Relationships between blood hemoglobin (Hb) levels (g/L) and clinicopathological characteristics in two colorectal cancer cohorts.

Variable	Cohort 1 (n=148)		Cohort 2 (n=208)	
(n, Cohort 1; n, Cohort 2)	Blood Hb, Mean (SD)	P value	Blood Hb, Mean (SD)	P value
**All Patients**	126.7 (17.3)		126.5 (17.4)	
**Age**				
<65 (n=59; n=71)	127.9 (17.1)	0.496	130.8 (17.8)	0.011
≥65 (n=89; n=137)	125.9 (17.5)		124.3 (16.9)	
**Sex**				
Male (n=80; n=110)	130.6 (19.0)	0.002	130.9 (18.0)	<0.001
Female (n=68; n=98)	122.0 (13.7)		121.6 (15.4)	
**Location of tumor**				
Proximal colon (n=48; n=75)	117.3 (17.5)	<0.001	115.9 (14.3)	<0.001
Distal colon (n=28; n=45)	123.1 (14.4)		126.4 (16.8)	
Rectum (n=72; n=88)	134.4 (14.6)		135.6 (15.0)	
**Preoperative radiotherapy or chemoradiotherapy in rectal cancer patients**				
No (n=40; n=51)	133.8 (16.2)	0.725	136.0 (15.9)	0.787
Yes (n=32; n=37)	135.0 (12.6)		135.1 (14.0)	
**WHO grade**				
Grade 1 (n=21; n=58)	131.1 (17.2)	0.169	124.6 (17.5)	0.035
Grade 2 (n=108; n=121)	127.0 (17.4)		128.9 (17.4)	
Grade 3 (n=18; n=28)	120.7 (15.2)		120.2 (16.3)	
**TNM Stage**				
Stage I (n=27; n=54)	139.0 (14.5)	<0.001	129.1 (17.9)	0.347
Stage II (n=54; n=59)	122.3 (16.6)		123.5 (17.7)	
Stage III (n=44; n=71)	128.3 (18.0)		127.5 (16.9)	
Stage IV (n=22; n=24)	119.2 (11.4)		125.2 (17.2)	
**Primary tumor**				
T1 (n=5; n=11)	149.8 (12.8)	<0.001	130.3 (13.3)	0.191
T2 (n=29; n=60)	135.7 (13.1)		129.5 (17.8)	
T3 (n=102; n=116)	124.4 (16.8)		125.7 (17.1)	
T4 (n=11; n=21)	115.6 (16.2)		120.8 (19.4)	
**Lymph node metastasis**				
N0 (n=85; n=115)	127.3 (17.6)	0.131	126.1 (17.8)	0.610
N1 (n=36; n=61)	130.2 (15.2)		128.2 (17.7)	
N2 (n=25; n=32)	121.2 (17.9)		124.7 (15.7)	
**Distant Metastasis**				
M0 (n=126; n=184)	127.8 (17.8)	0.004	126.7 (17.5)	0.686
M1 (n=22; n=24)	120.0 (11.7)		125.2 (17.2)	
**Infiltrative growth pattern**				
No (n=104; n=174)	126.7 (17.3)	0.870	127.3 (17.3)	0.114
Yes (n=43; n=34)	127.2 (17.3)		122.5 (17.9)	
**Lymphatic invasion**				
No (n=83; n=109)	126.9 (17.2)	0.964	127.1 (17.6)	0.601
Yes (n=61; n=99)	127.1 (17.6)		125.9 (17.4)	

**Blood vessel invasion**				
No (n=117; n=176)	126.6 (17.5)	0.574	127.2 (17.3)	0.199
Yes (n=27; n=32)	128.7 (16.9)		122.9 (17.8)	
**Mismatch repair (MMR) enzyme status**				
MMR Proficient (n=136; n=179)	127.3 (16.8)	0.239	128.3 (17.2)	<0.001
MMR Deficient (n=11; n=29)	120.9 (22.3)		115.8 (15.0)	
**BRAF VE1 immunohistochemistry**				
Negative (n=133; n=189)	127.5 (17.2)	0.058	127.2 (17.7)	0.082
Positive (n=14; n=19)	118.3 (17.0)		119.9 (13.3)	
**Modified Glasgow Prognostic Score (mGPS)**				
0 (n=118; n=151)	129.3 (17.4)	<0.001	128.3 (17.1)	0.006
1 (n=25; n=38)	118.2 (12.6)		121.2 (17.6)	
2 (n=5; n=3)	106.8 (3.1)		103.3 (9.2)	
**Mean corpuscular volume (MCV)**				
<80 (n=16; n=29)	103.7 (10.5)	<0.001	109.3 (11.0)	<0.001
80–100 (n=130; n=176)	129.6 (15.9)		129.2 (16.7)	
>100 (n=2; n=3)	125.0 (1.41)		134.0 (18.5)	

Table S4. Relationships between erythrocyte mean corpuscular volume (MCV) levels (fL) and clinicopathological characteristics in two colorectal cancer cohorts

**Variable**	**Cohort 1**		**Cohort 2**	
(n, Cohort 1; n, Cohort 2)	MCV, Mean (SD)	P value	MCV, Mean (SD)	P value
**All Patients**	88.6 (7.0)		86.6 (6.3)	
**Age**				
<65 (n=59; n=71)	88.7 (7.3)	0.937	86.4 (6.8)	0.643
≥65 (n=89; n=137)	88.6 (6.9)		86.8 (6.1)	
**Sex**				
Male (n=80; n=110)	89.8 (7.3)	0.028	86.8 (6.5)	0.636
Female (n=68; n=98)	87.3 (6.4)		86.4 (6.1)	
**Location of tumor**				
Proximal colon (n=48; n=75)	85.0 (8.2)	<0.001	84.1 (6.1)	<0.001
Distal colon (n=28; n=45)	88.5 (6.0)		85.8 (6.4)	
Rectum (n=72; n=88)	91.1 (5.3)		89.2 (5.4)	
**Preoperative radiotherapy or chemoradiotherapy in rectal cancer patients**				
No (n=40; n=51)	90.4 (5.0)	0.235	89.5 (5.2)	0.626
Yes (n=32; n=37)	91.9 (5.7)		88.9 (5.8)	
**WHO grade**				
Grade 1 (n=21; n=58)	91.2 (8.4)	0.189	86.0 (6.8)	0.174
Grade 2 (n=108; n=121)	88.1 (6.7)		87.3 (6.0)	
Grade 3 (n=18; n=28)	88.8 (6.9)		85.1 (6.6)	
**TNM Stage**				
Stage I (n=27; n=54)	92.2 (6.0)	0.005	88.4 (6.0)	0.022
Stage II (n=54; n=59)	87.2 (6.6)		84.8 (7.0)	
Stage III (n=44; n=71)	89.5 (7.8)		86.8 (5.5)	
Stage IV (n=22; n=24)	86.1 (5.8)		86.8 (6.7)	
**Primary tumor**				
T1 (n=5; n=11)	90.8 (7.1)	0.005	92.1 (5.4)	0.001
T2 (n=29; n=60)	92.6 (5.9)		88.1 (5.7)	
T3 (n=102; n=116)	87.6 (7.1)		85.6 (6.4)	
T4 (n=11; n=21)	87.2 (5.9)		85.1 (5.7)	
**Lymph node metastasis**				
N0 (n=85; n=115)	88.6 (6.8)	0.178	86.7 (6.9)	0.970
N1 (n=36; n=61)	90.3 (6.8)		86.5 (5.3)	
N2 (n=25; n=32)	86.9 (7.8)		86.6 (6.1)	
**Distant Metastasis**				
M0 (n=126; n=184)	89.1 (7.1)	0.071	86.6 (6.3)	0.934
M1 (n=22; n=24)	86.1 (5.8)		86.8 (6.7)	
**Infiltrative growth pattern**				
No (n=104; n=174)	88.9 (6.8)	0.618	86.9 (6.2)	0.235
Yes (n=43; n=34)	88.2 (7.5)		85.5 (7.0)	
**Lymphatic invasion**				
No (n=83; n=109)	89.0 (6.7)	0.504	86.9 (6.6)	0.550
Yes (n=61; n=99)	88.3 (7.4)		86.4 (6.0)	

**Blood vessel invasion**				
No (n=117; n=176)	88.5 (7.3)	0.410	86.9 (6.4)	0.215
Yes (n=27; n=32)	89.7 (5.5)		85.4 (5.9)	
**Mismatch repair (MMR) enzyme status**				
MMR Proficient (n=136; n=179)	88.7 (7.0)	0.617	87.1 (6.3)	0.021
MMR Deficient (n=11; n=29)	87.6 (7.2)		84.1 (5.6)	
**BRAF VE1 immunohistochemistry**				
Negative (n=133; n=189)	88.9 (6.9)	0.066	86.6 (6.4)	0.949
Positive (n=14; n=19)	85.3 (7.6)		86.7 (5.4)	
**Modified Glasgow Prognostic Score (mGPS)**				
0 (n=118; n=151)	89.1 (7.4)	0.292	87.3 (6.2)	0.016
1 (n=25; n=38)	86.9 (5.3)		84.7 (6.4)	
2 (n=5; n=3)	86.4 (3.5)		86.8 (6.3)	

Table S5. Relationships between different categories of anemia, mismatch repair (MMR) enzyme status, and BRAF VE1 immunohistochemistry in proximal colon

**Variable**	No anemia (n=39)	Microcytic anemia (n=28)	Normocytic anemia (n=56)	P value
**MMR enzyme status**				
MMR Proficient	29 (34.5%)	19 (22.6%)	36 (42.9%)	0.577
MMR Deficient	10 (25.6%)	9 (23.1%)	20 (51.3%)	
**BRAF VE1 immunohistochemistry**				
Negative	30 (31.3%)	23 (24.0%)	43 (44.8%)	0.885
Positive	9 (33.3%)	5 (18.5%)	13 (48.1%)	

Due to the small number of macrocytic anemia cases (n=2), macrocytic anemia category was not included in the analysis.

Table S6. Correlation between blood hemoglobin, erythrocyte mean corpuscular volume (MCV) and systemic inflammatory markers in the combined cohort.

	Unadjusted		Adjusted	
	Pearson r	p value	Beta	p value
**Blood hemoglobin**				
Serum CRP	-0.233	<0.001	-0.124	0.012
Serum Albumin	0.302	<0.001	0.253	<0.001
**Erythrocyte mean corpuscular volume**				
Serum CRP	-0.202	<0.001	-0.121	0.021
Serum Albumin	0.066	0.215	0.025	0.615

Serum CRP was logarithmically transformed because of positive skewness. The correlations were adjusted for tumor location based estimated tumor distance from anus, tumor stage variables (T1-2 vs. T3-4; N0 vs. N1-2; M0 vs. M1), and patient gender. Abbreviations: CRP: C-reactive protein; MCV: mean corpuscular volume.

Table S7. Correlation between blood hemoglobin levels and systemic inflammatory markers in Cohort 1.

	Unadjusted		Adjusted	
	Pearson r	p value	Beta	p value
**Systemic inflammatory markers**				
Serum CRP	-0.265	0.0012	-0.144	0.058
Serum Albumin	0.309	<0.001	0.279	<0.001
**Serum cytokines**				
IL-1RA	-0.177	0.031	-0.070	0.360
IL-4	0.005	0.947	0.078	0.291
IL-6	-0.210	0.011	-0.049	0.527
IL-7	-0.070	0.401	-0.023	0.760
IL-8	-0.346	<0.001	-0.227	0.009
IL-9	0.014	0.864	0.092	0.210
IL-12	-0.115	0.164	0.022	0.764
IFN-γ	-0.023	0.780	0.058	0.433
CXCL10	-0.172	0.037	-0.090	0.225
CCL2	0.062	0.456	0.101	0.176
CCL4	-0.113	0.172	-0.096	0.187
CCL11	0.055	0.508	0.001	0.985
PDGF-BB	-0.029	0.723	0.003	0.972

Serum CRP and serum cytokines were logarithmically transformed because of positive skewness. The adjusted correlations were calculated with multiple linear regression. The correlations were adjusted for tumor location based estimated tumor distance from anus, tumor stage variables (T1-2 vs. T3-4; N0 vs. N1-2; M0 vs. M1), and patient gender. Abbreviations: CCL: Chemokine (C-C motif) ligand; CRP: C-reactive protein; CXCL: Chemokine (C-X-C motif) ligand; IFN: interferon IL: interleukin; MCV: mean corpuscular volume; PDGF: Platelet-derived growth factor.

Table S8. Correlation between different classes of anemia and systemic inflammatory markers.

	No anemia	Microcytic anemia	Normocytic anemia	P value	P value (microcytic anemia vs normocytic anemia or no anemia)	P value (normocytic anemia vs microcytic anemia or no anemia)
**Combined cohort**						
CRP (mg/L)	2.0 (0.8-5.7)	5.0 (1.0-9.8)	5.3 (0.9-13.7)	0.002	0.269	0.003
Albumin (g/L)	43 (41-45)	43 (40-44)	41 (38.5-44)	<0.001	0.603	<0.001
**Cohort 1**						
CRP (mg/L)	1.8 (0.8-4.7)	1.8 (0.6-8.2)	6.9 (0.9-18.1)	0.053	0.792	0.015
Albumin (g/L)	43 (41-46)	43 (39.8-44)	41.5 (37-45)	0.008	0.326	0.007
IL-1RA (pg/mL)	60.2 (37.9-89.1)	67.2 (38.6-132.8)	63.4 (38-99.2)	0.253	0.132	0.616
IL-4 (pg/mL)	0.8 (0.7-1)	0.9 (0.6-1.2)	0.9 (0.7-1.1)	0.571	0.694	0.356
IL-6 (pg/mL)	4.4 (3.2-6.9)	7.2 (3.4-10.3)	6.5 (4.1-11.9)	0.017	0.281	0.015
IL-7 (pg/mL)	5.6 (4.2-7.5)	5.5 (4.4-11.6)	5.9 (3.8-8)	0.857	0.565	0.996
IL-8 (pg/mL)	10.7 (8.6-13.6)	13.8 (11.2-25.5)	15.2 (10-25.1)	<0.001	0.100	0.001
IL-9 (pg/mL)	8.7 (6-13.4)	9.6 (6.2-13.4)	8.1 (4.7-16.6)	0.968	0.859	0.870
IL-12 (pg/mL)	27.2 (13.2-37.7)	32.5 (17.9-43)	33.5 (17-46.9)	0.406	0.643	0.226
IFN-γ (pg/mL)	28.9 (23.6-40.1)	31 (23.4-49.6)	33.7 (23.1-46.4)	0.470	0.492	0.357
CXCL10 (pg/mL)	894.6 (659.6-1215.2)	967.6 (751.8-1176.8)	986.1 (757-1452.2)	0.373	0.982	0.150
CCL2 (pg/mL)	17.2 (12.2-23.7)	15.9 (10.8-24.9)	18.7 (11.4-28.4)	0.681	0.415	0.667
CCL4 (pg/mL)	62.3 (51-83.1)	61 (50.2-88.1)	68.1 (47.8-86)	0.718	0.945	0.410
CCL11 (pg/mL)	131.2 (91.7-174.3)	139.3 (89-193.4)	131.1 (95.9-185.6)	0.983	0.915	0.825
PDGF-BB (pg/mL)	8480 (6021.6-11360.5)	8263.3 (5806.8-15191.2)	8109.6 (4842.8-11261.9)	0.753	0.579	0.549
**Cohort 2**						
CRP (mg/L)	2.7 (0.7-6.1)	5.1 (1.6-17.0)	4.6 (0.9-12.4)	0.026	0.108	0.090
Albumin (g/L)	43.0 (41.0-45.0)	43.0 (41.0-45.0)	41.5 (39.0-44.0)	<0.001	0.852	<0.001

Abbreviations: CCL: Chemokine (C-C motif) ligand; CRP: C-reactive protein; CXCL: Chemokine (C-X-C motif) ligand; IFN: interferon IL: interleukin; MCV: mean corpuscular volume; PDGF: Platelet-derived growth factor.
